# Nephrocalcinosis: unveiling renal tubulopathies in the genomic era

**DOI:** 10.1590/2175-8239-JBN-2025-0225en

**Published:** 2026-01-30

**Authors:** Elenice Andrade Milhomem Ayoub, Maria Helena Vaisbich, Daniel Ribeiro Rocha, Bruno Pellozo Cerqueira, Ana Cristina Carvalho de Matos, Igor Gouveia Pietrobom, Ita Pfeferman Heilberg

**Affiliations:** 1 Universidade Federal de São Paulo, Divisão de Nefrologia, São Paulo, SP, Brazil.

**Keywords:** Nephrocalcinosis, Nephrolithiasis, Tubulopathies, Renal Tubular Acidosis, Primary Hyperparathyroidism.

## Abstract

**Introduction:**

The diagnosis of nephrocalcinosis (NC) is challenging due to difficulties in radiologic detection, clinical heterogeneity, and its broad etiological spectrum that includes both genetic and non-genetic causes. This study aimed to uncover, in a pioneering manner, the distinct NC etiologies in a Brazilian cohort according to age of onset.

**Methods:**

This retrospective cross-sectional study was based on 96 medical records of outpatients (66 adults and 30 children) with NC, who had followed a comprehensive investigation.

**Results:**

Distal renal tubular acidosis was the leading cause of NC in both children (43.3%) and adults (22.7%), followed by other tubulopathies mostly in children (36.7%), and primary hyperparathyroidism in adults (19.7%). Pediatric patients exhibited more electrolyte disorders (47%), failure to thrive (50%), and sensorineural hearing loss (23%) than adults. Conversely, adults presented more associated nephrolithiasis (73%), low back pain (42%), and lower estimated glomerular filtration rate than children (85 *versus* 106 mL/min/1.73m^2^). According to phenotype, genetic suspicion had been raised in 96.7% of children and 57.6% of adults. Nevertheless, the availability of genetic tests was low in the whole cohort.

**Conclusion:**

This study represents a novel contribution to the national scientific landscape, revealing a limited access to genetic tests for patients with nephrocalcinosis, likely due to cost and availability constraints in the public healthcare system, and showed that a clinically oriented laboratory protocol may provide more precise indications. Notably, a phenotype suggestive of monogenic disease among 57.6% of adults with NC justifies the need for more frequent genetic investigations in this group.

## Introduction

Nephrocalcinosis (NC) is a progressive and often clinically silent pathological condition characterized by calcium phosphate or calcium oxalate deposits in the renal parenchyma^1^. NC is not necessarily associated with nephrolithiasis (NL), and although they represent distinct entities, both are closely interconnected, making their radiographic distinction challenging in some cases^2,3^. Few studies have systematically compared different radiological methods. In a previous study by our group among adults, a combination of CT scans with either US or kidney-ureter-bladder (KUB) X-ray yielded the most accurate results^4^. NC is a common sign of an underlying disease and does not represent an etiological diagnosis itself. It carries high morbidity, including the risk of progression to chronic kidney disease (CKD), because of crystal-induced tubular injury, that triggers inflammatory and fibrotic pathways^5,6,7^. In adults, the most commonly reported causes are primary hyperparathyroidism (PHPT) and distal renal tubular acidosis (dRTA)^1,2^, whereas in pediatric patients, in addition to dRTA, other anatomical abnormalities and genetic diseases are more frequent^8^. Presentation at a young age, family history of an affected individual, extrarenal manifestations, or a history of consanguinity in the family are often suggestive of hereditary causes of NC^9,10^. The Brazilian population is characterized by a high degree of admixture and diverse ancestry. This genetic diversity offers unique opportunities to uncover novel variants and distinct clinical presentations not observed in more genetically homogeneous populations^11^.

The present study aimed to identify the underlying etiologies of NC through clinical presentation, laboratory characteristics and genetic testing, according to the age of radiologic detection.

## Methods

This was a retrospective cross-sectional study based on data obtained from medical records of patients referred to a single ambulatory center. Patients were categorized into pediatric (<18 years old) or adult (≥18 years old) groups based on the age at radiological diagnosis of NC. The inclusion criteria comprised having radiologic diagnosis of NC by unenhanced CT scans and/or renal US upon admission, metabolic workup from at least one 24-hour urine sample or spot urine (in case of poor sphincter control, in small children), and serum biochemistry/hormonal data. Demographic and anthropometric parameters were collected, as well as manifestations at the first medical appointment like abdominal or low back pain, concomitant presence of NL, history of recurrent urinary tract infections (UTI) or previous urological procedures, extrarenal manifestations (deafness, muscle weakness, failure to thrive), electrolyte disorders, and comorbidities (arterial hypertension and diabetes mellitus).

The standardized routine investigation protocol consisted of calcium, sodium, potassium, citrate, oxalate, phosphorus, uric acid, magnesium, and cystine measured in 24-hour urinary samples. Diagnostic criteria for metabolic urinary disorders were defined in adults as described elsewhere^12^. In children who collected spot urine samples, metabolic alterations were diagnosed using reference values described by Hoppe and Higueras^13^. Measurements of morning 12-hour fasting urinary pH in spot urine samples obtained simultaneously with venous blood pH and plasma bicarbonate were considered for diagnosing dRTA. In the absence of spontaneous acidosis, patients were further subjected to an ammonium chloride (NH_4_Cl) loading test to detect incomplete forms of dRTA, as described previously^4^. Other serum parameters determined were urea, creatinine, potassium, magnesium, uric acid, calcium, phosphorus, parathyroid hormone (PTH), 25-hydroxyvitamin-D (25(OH)D), and 1,25 (OH)_2_ vitamin D. Creatinine was determined by IDMS (isotope dilution mass spectrometry) and the estimated glomerular filtration rate (eGFR) was calculated using the updated Schwartz formula for children and CKD-EPI (2021) for adults. Genetic tests, whenever available, mainly consisted of gene panels or whole exome sequencing (WES). The interpretation of the pathogenicity of a variant was based on ACMG (American College of Medical Genetics and Genomics) criteria^14^, and the correspondence to phenotype was then evaluated.

### Statistical Analysis

Variable distributions were evaluated using the Shapiro-Wilk test. Normally distributed data were expressed as mean ± standard deviation (SD) and non-normally distributed as median [interquartile range]. Differences between groups were assessed using the T-test or Mann-Whitney according to the variable’s distribution. Categorical variables, presented as n (%), were compared using a chi-square test. Analyses were performed using JAMOVI, and p values <0.05 were considered statistically significant.

## Results

Ninety-six (96) patients, 66 adults and 30 children, with a radiologic diagnosis of NC at admission were included. Most adult patients, 60 out of 66 (91%) had NC based on ultrasound (US) and confirmed by CT scans while 6 were diagnosed by US only. Among children, the radiological diagnosis was based on US and confirmed by CT scans in 14 out of 30 cases (47%), 14 were diagnosed by US only and 2 were diagnosed by CT scan only. Clinical and laboratory characteristics are described in Table 1. In adults, there was a significantly higher distribution of females, more association with NL, and lower median eGFR than in children. The number of adults with stage 2 CKD or higher significantly outnumbered pediatric cases. Diabetes and arterial hypertension were not seen in the pediatric group. Although apparently longer for adults, the median diagnosis delay was not statistically different between groups.

**Table 1:** Clinical and laboratory characteristics of all patients with radiological nephrocalcinosis (NC) at admission

	< 18 years (n = 30)	≥ 18 years (n = 66)	p-value
Age at first signs/symptoms, years	4.5 [0–8.5]	27.0 [18–35]	
Female sex, n (%)	15 (50.0%)	49 (74.2%)	**0.02**
Diagnostic delay, years	4.5 [1.0–9.8]	8.0 [2.0–15.0]	0.14
Associated nephrolithiasis	13 (43.3%)	48 (72.7%)	**0.01**
Urological procedures	8 (26.7%)	30 (45.5%)	0.08
Diabetes mellitus	0	4 (6%)	N/A
Arterial hypertension	0	11 (17%)	N/A
Hematuria	11/23 (47.8%)	17/49 (34.7%)	0.29
Leukocyturia	9/23 (39.1%)	25/48 (52.1%)	0.34
eGFR (mL/min/1.73 m^2^)	106 [91–123]	85 [59–113]	**0.01**
stage 1 CKD	23 (77.0%)	32 (48.5%)	**0.01**
stages 2 through 5 CKD	7 (23.0%)	34 (51.5%)	**0.05**

Abbreviations – eGFR: estimated Glomerular Filtration Rate; CKD: Chronic Kidney Disease; N/A: Not applicable.

Notes – Median [IQR] [25%–75%]; number of cases/total number of cases.

**Figure 1:**
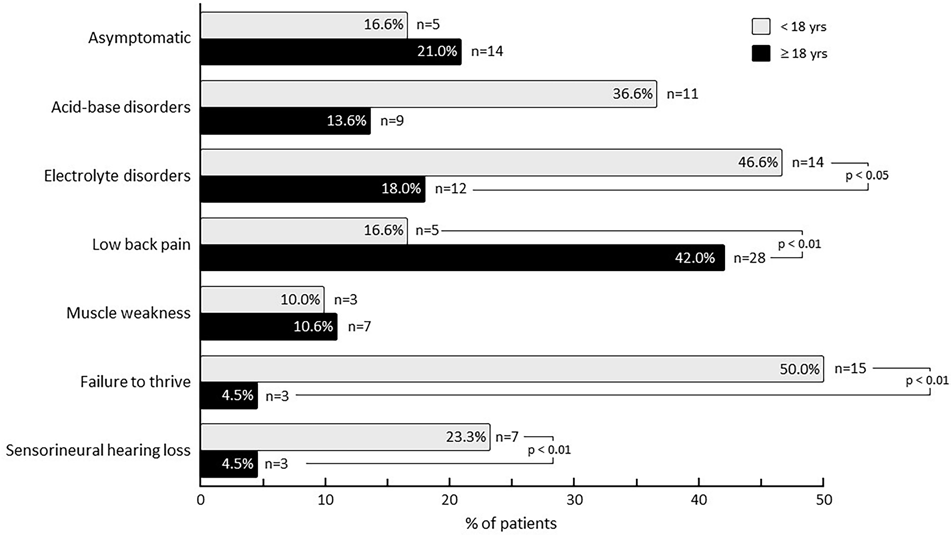
Main clinical and laboratory characteristics of patients at diagnosis of radiologic nephrocalcinosis (NC).

Figure 1 shows the main manifestations observed in pediatric and adult groups at radiologic diagnosis of NC. The percentage of asymptomatic cases was not statistically different between children and adults. Of the symptomatic cases, failure to thrive, sensorineural hearing loss, and electrolyte disorders were significantly more frequent in children and low back pain was significantly more frequent in adults.

### Etiology of NC Based on Initial Phenotype

Table 2 shows the diagnostic hypothesis derived from clinical, laboratory, and kidney imaging data during NC detection. The cases were categorized into three groups: not suggestive of genetic origin, suspected monogenic origin, and undefined causes where no suggestive phenotype was identified.

**Table 2:** Etiology of nephrocalcinosis (NC) based on initial phenotype

	< 18 years (n = 30)	≥ 18 years (n = 66)	p-value
Phenotype not suggestive of genetic origin (n = 25)			
Number of cases	**1/30 (3.3%)**	**24/66 (36.4%)**	**< 0.001**
PHPT	0	13 (54.0%)	
Drug-induced^#^	0	5 (21.0%)	
MSK	1 (3.3%)	2 (8.3%)	
Post-surgical hypoparathyroidism	0	2 (8.3%)	
Complete dRTA + Sjogren syndrome	0	2 (8.3%)	
Phenotype suggestive of monogenic origin (n = 67)			
Number of cases	**29/30 (96.7%)**	**38/66 (57.6%)**	**< 0.001**
Complete dRTA	10 (34.5%)	10 (26.2%)	
Incomplete dRTA	3 (10.4%)	5 (13.0%)	
HHRH carrier	3 (10.4%)	1 (2.6%)	
ADH	2 (6.8%)	0	
FHHNC	2 (6.8%)	0	
Dent disease	4 (13.8%)	0	
Bartter	0	3 (8.0%)	
Lowe	0	1 (2.6%)	
PH1	1 (3.5%)	1 (2.6%)	
Isolated hypercalciuria	4 (13.8%)	11 (29.0%)	
Isolated hypocitraturia	0	6 (16.0%)	
Undefined causes			
	**0**	**4/66 (6.0%)**	**N/A**

Abbreviations – dRTA: distal Renal Tubular Acidosis; FHHNC: Familial Hypomagnesemia with Hypercalciuria and Nephrocalcinosis; HHRH: Hereditary hypophosphatemic rickets with Hypercalciuria; ADH: Autosomal Dominant Hypocalcemia; MSK: Medullary Sponge Kidney; IIH type 2: Idiopathic Infantile Hypercalcemia type 2; PHPT: primary Hyperparathyroidism; PH1: Primary Hyperoxaluria type 1; N/A: Not applicable.

Notes – #Drugs included furosemide, anabolic steroids and corticosteroids.

Based on the data presented in Table 2, only one child (3.3%) exhibited a phenotype not suggestive of a monogenic origin of NC compared to 36.4% among adults. Nevertheless, the proportion of adults exhibiting a phenotype suggestive of monogenic disease was still significant (57.6%).

Distal RTA, including complete and incomplete forms, was the predominant cause of NC in both groups. PHPT was the second most prevalent diagnosis in adults (not evidenced in children) as well as drug-induced NC. Medullary sponge kidney (MSK) was rarely diagnosed in children or adults. Other rare hereditary tubulopathies were predominantly identified in children, but some of them were only diagnosed later in adulthood. PH1, a severe genetic metabolic disease, was detected in one case in each group, both in patients with stage 5 CKD. Isolated hypercalciuria or hypocitraturia was more frequent in adults. In four adults (6.0%), the cause of NC remained undefined.

### Genetic Testing

Genetic test results were not available in all cases, having been performed in 29 patients (16 children and 13 adults), whose findings are presented in Table 3. Genetic diagnosis based on suspicion of a monogenic origin of NC was disclosed in 55.2% of pediatric cases (16/29) versus 30.9% (13/42) of adults, p = 0.05. In the whole cohort, genetic analysis resulted in successful diagnosis in 22 of 29 tests, achieving a diagnostic rate of 76.0%, confirming the phenotypic diagnosis in 65.5% (19/29) of cases and changing it in 10.4% (3/29). In the pediatric group, clinical diagnosis was confirmed in 68.7% (11/16) and modified at 12.5% (2/16) of the cases, yielding a successful rate of 81.2% (13/16). In adults, the test confirmed the clinical hypothesis in 61.5% (8/13) and changed it in 7.7% (1/13), achieving a successful rate of 69.2% (9/13). The diagnosis was revised in 2 pediatric cases: a 13-year-old girl initially suspected of having Dent disease (case P11) and an 11-year-old girl suspected of idiopathic hypercalciuria (case P12) were both ultimately diagnosed with idiopathic infantile hypercalcemia type 2 (IIH type 2) through genetic testing. Additionally, the diagnosis was revised in 1 adult female patient initially diagnosed with idiopathic hypercalciuria (case A8). The genetic testing demonstrated a variant of uncertain significance (VUS) in *SLC34A3*, which explained the clinical phenotype, hence being considered a disease-related variant. Similarly, for cases P6 and A7, the variant in *SLC34A3*, classified as VUS according to ACMG criteria, was also later considered a disease-related variant. In 24.1% (7/29) of patients, genetic testing did not contribute to diagnostic elucidation: four adults, including two patients (cases A12 and A13) with undefined initial diagnostic hypothesis, 1 case of idiopathic hypercalciuria (A11) with no variant detected through genetic testing, and 1 case of incomplete dRTA (A6) with detection of a likely benign variant in *OXGR1*; three pediatric patients, including 1 case of suspected incomplete dRTA (P4) with identification of a heterozygous VUS in *GRHPR*, 1 case of suspected Dent disease with identification of biallelic variants in *CUBN*, which could justify proteinuria but not hypercalciuria, and one patient with idiopathic hypercalciuria with a heterozygous pathogenic variant in *COL4A3*.

**Table 3:** Genetic Test Results

Pt.	Sex (M/F) Age (yrs)	Clinical suspicion	Gene zygosity	Nucleotide; amino acid	Classification (ACMG)	Confirmed diagnosis	Changed diagnosis	Genetic final diagnosis	Additional findings (gene/state/nucleotide; protein/classification)
Pediatric Cohort (n = 16)									
P1	M 7	dRTA	*SLC4A1* Het	c.2661_2680del; p.Ala888Leufs*2	Pathogenic	X	----	dRTA	N/A
P2	M 10	dRTA	*SLC4A1* Het	c.2661_2680del; p.Ala888Leufs*2	Pathogenic	X	----	dRTA	N/A
P3	F 14	idRTA	*SLC4A1* Het	c.1825G>A;p.Gly609Arg	Pathogenic	X	----	Incomplete dRTA	N/A
P4	M 5	idRTA	no NC-related gene	N/A	N/A	----	----	Not yet defined	GRHPR/Het c.149C>T:p.Ala50Val/VUS
P5	M 1	HHRH	*SLC34A3* Het	c.1217G>T;p.Gly406Val	Pathogenic	X	----	HHRH (carrier)	N/A
P6	F 2	HHRH	*SLC34A3* Het	c.232G>A;p.Gly78Arg	VUS^#^	X	----	HHRH (carrier)	N/A
P7	F 7	HHRH	*SLC34A3* Het	c.1217G>T;p.Gly406Val	Pathogenic	X	----	HHRH (carrier)	N/A
P8	M 4	Dent	*CLCN5* Hem X-linked	c.2151C>T; p.Arg718Thr	Pathogenic	X	----	Dent disease 1	N/A
P9	M 7	Dent	*OCRL* Hem X-linked	c.217_218delTT; p.Leu73Aspfs*2	Pathogenic	X	----	Dent disease 2	N/A
P10	F 4	Dent	no NC-related gene	N/A	N/A	----	----	Not yet defined	*CUBN* / Compound Het c.8599G>C;p.Val2867Leu/ VUS c.9053A>C; p.Tyr3018Ser/Likely pathogenic
P11	F 13	Dent	*SLC34A1* Het	c.460_480dup; p.Ile154_Val160dup	Pathogenic	----	X	IIH type 2	N/A
P12	F 11	IH	*SLC34A1* Het	c.644G>A; p.Arg215Gln	Pathogenic	----	X	IIH type 2	*SLC34A3*/Het c.1765G>A; p.Glu589Lys/Likely benign
P13	M 5	PH	*AGXT* Hom	exon 7 del	Pathogenic	X	----	PH1	N/A
P14	M 0.8	FHHNC	*CLDN16* Hom	c.325_c918*?; E2_E5del	Pathogenic	X	----	FHHNC	N/A
P15	F 6	ADH	*CASR* Het	c.2833T>C; p.Phe821Leu	VUS^#^	X	----	ADH	N/A
P16	F 17	IH	no NC-related gene	N/A	N/A	----	----	Not yet defined	*COL4A3/*Het c.4510T>C:p.Phe1504Le/VUS Adult Cohort (n = 13)
Adult Cohort (n = 13)									
A1	F 18	dRTA	*SLC4A1* Het	c.2716G>T;p.Glu906*	Pathogenic	X	----	dRTA	N/A
A2	F 23	dRTA	*SLC4A1* Het	c.2381A>G;p.Tyr794Cys	VUS^#^	X	----	dRTA	*SLC3A1*/Het c.1047C>A; p.Asp349Glu/VUS
A3	M 23	dRTA	*ATP6V1B1* Het	c.1181G>A;p.Arg394GIn	VUS^#^	X	----	dRTA	N/A
A4	M 31	dRTA	*ATP6V1B1* Het	c.1181G>A;p.Arg394GIn	VUS^#^	X	----	dRTA	N/A
A5	M 64	dRTA	*ATP6V1B1* Het	c.1181G>A;p.Arg394GIn	VUS^#^	X	----	dRTA	N/A
A6	M 27	idRTA	*OXGR1* Het	c.566C>T;p.Ser189Leu	Likely benign (*novel*)	----	----	Not yet defined	N/A
A7	M 41	HHRH	*SLC34A3* Het	c.232G>A;p.Gly78Arg	VUS	X	----	HHRH (carrier)	N/A
A8	F 21	IH	*SLC34A3* Het	c.413C>T / p.Ser138Phe	VUS	----	X	HHRH (carrier)	*GRHPR*/Het c296G>A; p.Arg99Gln / VUS
A9	M 37	PH	*AGXT* Compound Het	c.508G>A;p.Gly170Arg - c.283G>A;p.Glu95Lys	Pathogenic/ Likely pathogenic	X	----	PH1	N/A
A10	F 42	BS	*KCNJ1* Het	c.657C>A;p.Ser219Arg	Likely pathogenic	----	----	BS type 2 (carrier)	N/A
A11	F 35	IH	negative	N/A	N/A	N/A	N/A	Not yet defined	N/A
A12	F 27	No NC-related disease	negative	N/A	N/A	N/A	N/A	Not yet defined	N/A
A13	F 29	No NC-related disease	negative	N/A	N/A	N/A	N/A	Not yet defined	N/A

Abbreviations – Het: heterozygous; hem: hemizygous; hom: homozygous; dRTA: incomplete distal renal tubular acidosis; idRTA; FHHNC: Familial Hypomagnesemia with Hypercalciuria and Nephrocalcinosis; HHRH: Hereditary hypophosphatemic rickets with Hypercalciuria; ADH: autosomal dominant hypoparathyroidism; IH: idiopathic hypercalciuria; IIH type 2: Idiopathic Infantile Hypercalcemia type 2; BS: Bartter syndrome; PH1: Primary Hyperoxaluria type 1; VUS: variant of unknown significance; N/A: Not applicable.

Notes – ^#^genetic variant considered as disease-related variant.

### Final Diagnosis

**Figure 2:**
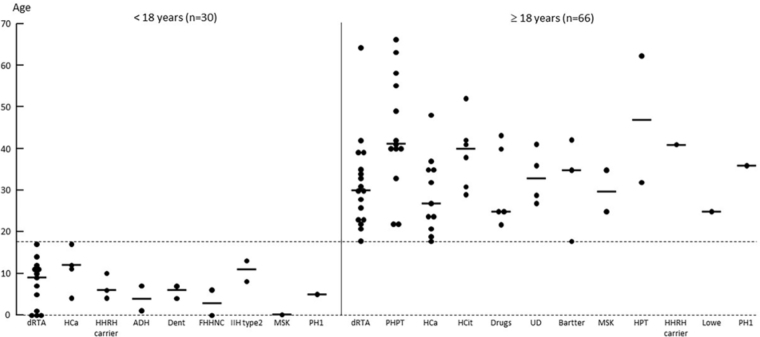
Nephrocalcinosis (NC) etiology (descending order of frequency) according to individual ages (o) of radiologic diagnosis. Horizontal lines indicate median values.

The individual and median age distributions at NC diagnosis according to the etiologies are shown in Figure 2.

Abbreviations – dRTA: Distal Renal Tubular Acidosis; HCa: Idiopathic Hypercalciuria; HCit: Hypocitraturia; HHRH: Hereditary hypophosphatemic rickets with Hypercalciuria; ADH: Autosomal Dominant Hypocalcemia; FHHNC: Familial Hypomagnesemia with Hypercalciuria and Nephrocalcinosis; IIH type 2: Idiopathic Infantile Hypercalcemia type 2; MSK: Medullary Sponge Kidney; PHPT: Primary Hyperparathyroidism; PH1: Primary Hyperoxaluria type 1; HPT: Post-surgical Hypoparathyroidism; UD: Undefined.

## Discussion

Clinical manifestations of NC can be heterogeneous, ranging from asymptomatic cases to renal or systemic alterations whose characteristics vary depending on the underlying etiology and timing of diagnosis. In any case, underlying monogenic kidney diseases are more common than in nephrolithiasis^7,10,15^. To the best of our knowledge, there is no other series of NC etiologies across all ages in Brazil. Moreover, Brazil is underrepresented in global genetic databases related to many diseases^16^. Therefore, we aimed to retrospectively identify the diverse NC etiologies and compare the clinical and biochemical profiles of adult and pediatric patients at the time of radiological diagnosis. Notably, significant differences in etiology were observed between the two groups, except for dRTA, which was the leading cause in both. Primary hyperparathyroidism was evidenced only in adults, and genetic diseases mostly in children. However, genetic testing had been more often performed in the latter.

In the present series, adults presented with a lower eGFR at diagnosis than children, possibly due to delayed NC diagnosis or the higher prevalence of prior urological procedures, but the difference was not statistically significant. Comorbidities and associated NL in adults (72.7%), as seen in other studies^17,18^ might also have contributed to reduced renal function. Importantly, failure to thrive and deafness were frequent in children, likely reflecting the higher prevalence of hereditary tubulopathies^19,20,21^.

### Distal Renal Tubular Acidosis (dRTA)

According to clinical and laboratory data, both complete and incomplete forms of dRTA were the most common cause of NC in this cohort, identified in 43.3% of children and 22.7% of adults. Our results align with a previous report, which identified dRTA as the most frequent cause of NC in a large cohort of 375 patients^1^ without distinguishing age. In children, rates of dRTA in NC in the literature vary from 5.6% to 34%^17,19,22^. In adults, an old report by Brenner et al.^23^ based on abdominal radiographs identified NC in 56% of cases of dRTA, but data specifying rates of dRTA as an etiology of NC in adults are still scarce^24^. The present findings emphasize the relevance of our large cohort of adults with NC revealing dRTA.

Regarding the molecular spectrum, dRTA was the leading genetic diagnosis in our cohort (8/22; 36.4%), encompassing all canonical genes (*SLC4A1*, *ATP6V0A4*, *ATP6V1B1*). Similar dRTA frequencies were reported by series in adults^25^ and children^26^, whereas markedly lower dRTA rates were described by other investigators^10,21,27,28^. As several of these studies were conducted in a tertiary referral center, different populations and pre-laboratory workup might have contributed to variation in diagnostic yields and etiologic distributions.

In the present study, despite the strong phenotypegenotype concordance, we recognize that genetic testing should be recommended to all patients with a clinical suspicion of primary dRTA to refine prognosis and management, such as evaluating extra-renal manifestations and performing family screening, given that inheritance may be autosomal recessive or dominant^24,29^. For example, patient A4 (proband; Table 3) carried a heterozygous *ATP6V1B1* variant (c.1181G>A;p.Arg394Gln) associated with a very severe presentation of NC and dRTA. Segregation analysis identified the same variant in his brother (A3) and father (A5), both with similar phenotypes, while genetic testing was negative in his mother and another brother without clinical manifestations or NC. However, renal acidification defects and NL in heterozygous carriers have been previously described in the B1 unit of this gene^30,31,32^. Therefore, although this variant has been initially considered a VUS by ACMG classification, the correlation with the phenotype suggests it to be a disease-related variant.

### Primary Hyperparathyroidism (PHPT) and other Adult-Specific Etiologies

PHPT emerged as the second most common etiology among adults (54%), consistent with prior reports^33,34^. In contrast, no pediatric cases of PHPT were identified, underscoring the age-related differences in NC etiology. Medullary sponge kidney (MSK), the third most common cause mentioned by Shavit et al.^1^, was less frequent in our cohort. This discrepancy may reflect diagnostic limitations, as confirmation via intravenous urography or contrast-enhanced CT was not systematically performed.

### Genetic Variants in *SLC34A1* and *SLC34A3*

Another significant genetic finding from this Brazilian cohort was the high rate of disease-related heterozygous variants in *SLC34A1* and *SLC34A3* (31.8%), which encode the cotransporter sodium-phosphate, NaPi-2a and NaPi-2c, respectively. Several other investigators reported such variants in 10%^25^, 11.1%^10^, 23.9%^28^, 35.3%^21^, and 60%^5^ of cases, in association with hypercalciuria and nephrocalcinosis. We identified heterozygous variants in the *SLC34A3* gene (Table 3) in four patients (P5, P6, P7, A7), supporting its potential contribution to NC/NL phenotypes, even in the form of a VUS. The *SLC34A3* variant c.413C>T; p.Ser138Phe, in case A8, previously reported in hereditary hypophosphatemic rickets with hypercalciuria^20,35,36^, demonstrated reduced sodiumdependent phosphate cotransporter activity, suggesting its pathogenic role. Similarly, *SLC34A3* variant c.232G>A;p.Gly78Arg classified as VUS in cases P6 and A7 were considered disease-related based on phenotypic correlation as previously described^37^. With respect to modifying final diagnosis, the molecular detection of variants in the *SLC34A1* gene in patients P11 and P12 (previously suspected to have Dent disease and idiopathic hypercalciuria) changed the management. Given that approximately 50% of patients respond to phosphate supplementation therapy thiazides are contraindicated, and the renal prognosis is different concerning progression to CKD^37^.

### Other Etiologies and the role of Genetictesting

 Among other NC causes, primary hyperoxaluria type 1 (PH1) and hypoparathyroidism were identified in both groups. Uncovering these diagnoses emphasizes the need for prompt and thorough investigation, as some of these conditions can rapidly deteriorate renal function. PH1 is an example of a severe genetic disorder that, if left untreated, results in poor renal outcomes^6,38^, particularly considering that new specific treatment based on interference RNA is available^39^.

In summary, only a few published series have specifically evaluated NC and its etiologies. Moreover, most international cohorts usually do not report clinical, laboratory, and genetic data from NC at the time of screening^5,10,21,25,27,28,40^ separately from nephrolithiasis (NL), which complicates comparisons with the present study. Considering particularly NC (isolated or associated to NL), the disclosure of positive genetic testing in the present study was 76.0% compared to 33.3 to 84.0% in such series. However, the presence of monogenic disease in our child cohort was 81.2%, in agreement with 84% reported by Huang et al.^26^, although higher than 40% as found by Braun et al.^27^ and Gefen et al.^5^. Among adults, our rates of monogenic disease in NC reached 69.2%, compared with 83.3% described in a smaller recent sample of 12 adult patients^25^. Nevertheless, cross-cohort comparisons are easily biased by differences in referral patterns, inclusion criteria, and analytic pipelines (panel vs. exome, depth, CNV (copy number variation) calling, evolving ACMG), which shift diagnostic yield and the apparent contribution of specific genes. Population structure also matters: our highly admixed cohort (European, African, Indigenous descent) alters allele frequencies and reveals under-catalogued variants, hampering interpretation^11^. Therefore, population-informed analyses, regional reference maps of NC etiologies, and equitable access to comprehensive genetic testing are essential.

### Diagnostic and Prognostic Implications

In patients with a strong suspicion of monogenic disease, especially if relatives share the same phenotype, when the condition is potentially modifiable and amenable to specific personalized therapy, or when extrarenal manifestations warrant evaluation, genetic testing should be prioritized whenever feasible, as it can establish a definitive diagnosis and guide targeted treatment, as illustrated above. Moreover, molecular analysis is used in genetic counseling as well. However, as genetic testing is still unavailable in many centers that treat these patients, this study suggested that applying a rational routine investigation protocol may help define more precise diagnoses. Although monogenic diseases were suspected in almost all children, the proportion of adults exhibiting a phenotype suggestive of monogenic disease was still significant (57.6%), especially considering the high number of PHPT patients in this cohort. Such observation suggests that genetic testing should be employed more frequently in adults than it is currently.

### Limitations

The limitations of the present study are its retrospective and single-center design, missing values of laboratory tests from the medical records, and the lack of genetic tests for all patients. Correction for multiple comparisons was not applied in the present series due to the heterogeneity of etiologic diagnoses in each group.

## Conclusions

This study confirmed that NC is a common sign of an underlying kidney or systemic disease and it does not represent an etiological diagnosis itself. It carries high morbidity, including the risk of progression to CKD. The present findings reinforce that NC strongly suggests an underlying genetic disorder with renal involvement, especially in children, but that many adult cases may have a monogenic basis as well, justifying genetic testing also for adults. Given the limited access to genetic tests in our population, likely due to cost and availability in the public healthcare system, a structured clinical and laboratory evaluation may help define more accurately the indication of genetic testing across all age groups.

## Data Availability

All data will be made available on request.
