# Supplementary material for: Nephrocalcinosis: unveiling renal tubulopathies in the genomic era
Source: J Bras Nefrol. 2026 Jan 30;48(2):e20250225. doi: 10.1590/2175-8239-JBN-2025-0225en (PMC12857816; doi:10.1590/2175-8239-JBN-2025-0225en)
Supplement: Translation

# Translation: Nefrocalcinose: desvendando as tubulopatias renais na era genômica

**DOI:** 10.1590/2175-8239-JBN-2025-0225pt

**Authors:** Elenice Andrade Milhomem Ayoub, Maria Helena Vaisbich, Daniel Ribeiro Rocha, Bruno Pellozo Cerqueira, Ana Cristina Carvalho de Matos, Igor Gouveia Pietrobom, Ita Pfeferman Heilberg

**Affiliations:** 1 Universidade Federal de São Paulo, Divisão de Nefrologia, São Paulo, SP, Brasil.

**Keywords:** Nefrocalcinose, Nefrolitíase, Tubulopatias, Acidose Tubular Renal, Hiperparatireoidismo Primário

## Abstract

**Introdução:**

O diagnóstico de nefrocalcinose (NC) é desafiador devido a dificuldades na detecção radiológica, heterogeneidade clínica e amplo espectro etiológico, incluindo causas genéticas e não genéticas. O objetivo do estudo foi identificar, de forma pioneira, diferentes etiologias da NC em coorte brasileira, de acordo com a faixa etária de início.

**Métodos:**

Estudo transversal retrospectivo, análise de 96 prontuários ambulatoriais (66 adultos, 30 crianças) com NC, submetidos a investigação clínico-laboratorial abrangente.

**Resultados:**

Acidose tubular renal distal (ATRd) foi a principal causa de NC em crianças (43,3%) e adultos (22,7%), seguida por outras tubulopatias, mais frequentes em crianças (36,7%), e pelo hiperparatireoidismo primário (HPTP) em adultos (19,7%). Pacientes pediátricos apresentaram maior frequência de distúrbios eletrolíticos (47%), déficit de crescimento (50%) e perda auditiva neurossensorial (23%) comparados aos adultos. Por outro lado, adultos apresentaram maior ocorrência de nefrolitíase associada (73%), dor lombar (42%) e TFGe inferior à das crianças (85 versus 106 mL/min/1,73 m^2^). Conforme o fenótipo clínico, houve suspeita de etiologia genética em 96,7% das crianças e 57,6% dos adultos. A disponibilidade de testes genéticos foi baixa em toda a coorte.

**Conclusão:**

Este estudo contribui para o cenário científico nacional ao revelar acesso limitado a testes genéticos para pacientes com NC, provavelmente por custo e disponibilidade restrita no sistema público. Os achados demonstraram que protocolo laboratorial orientado clinicamente pode auxiliar na definição de indicações mais precisas para investigação genética. Fenótipo sugestivo de doença monogênica foi identificado em 57,6% dos adultos com NC, o que justifica necessidade de investigação genética mais frequente também nesse grupo.

## Introdução

A nefrocalcinose (NC) é uma condição patológica progressiva e clinicamente silenciosa, caracterizada por depósitos de fosfato de cálcio ou oxalato de cálcio no parênquima renal^1^. A NC não está necessariamente associada à nefrolitíase (NL) e, embora representem duas entidades distintas, ambas estão intimamente inter-relacionadas, o que torna a distinção radiográfica entre elas ainda mais desafiadora em alguns casos^2,3^. Poucos estudos compararam sistematicamente diferentes métodos radiológicos. Em um estudo anterior realizado por nosso grupo em adultos, a combinação de tomografia computadorizada (TC) com ultrassonografia (US) ou radiografia simples de abdome (Rx) apresentou melhor acurácia^4^. A NC é comumente um sinal de doença subjacente, não representando um diagnóstico etiológico em si,estando associada a alta morbidade, incluindo o risco de progressão para doença renal crônica (DRC), devido à lesão tubular induzida por cristais, que desencadeia vias inflamatórias e fibróticas^5,6,7^. Em adultos, as causas mais comumente relatadas são o hiperparatireoidismo primário (HPTP) e a acidose tubular renal distal (ATRd)^1,2^, enquanto, em pacientes pediátricos, além da ATRd, outras anormalidades anatômicas e doenças genéticas são frequentemente mais comuns^8^. O aparecimento em idade precoce, o histórico familiar, as manifestações extra-renais ou a presença de consanguinidade na família são, muitas vezes, sugestivos de causas hereditárias de NC^9,10^. A população brasileira é caracterizada por muita miscigenação e ancestralidade diversa. Essa diversidade genética oferece oportunidades únicas para identificar novas variantes e apresentações clínicas distintas, não observadas em populações geneticamente mais homogêneas^11^.

O presente estudo teve como objetivo identificar as etiologias subjacentes da NC por meio da análise da apresentação clínica, das características laboratoriais e dos testes genéticos, de acordo com a idade em que a NC foi detectada radiologicamente.

## Métodos

Este é um estudo transversal e retrospectivo baseado em dados obtidos de prontuários médicos de pacientes encaminhados a um único centro ambulatorial. Os pacientes foram categorizados em grupos pediátrico (<18 anos) ou adulto (≥18 anos), com base na idade ao diagnóstico radiológico de NC. Os critérios de inclusão compreenderam a disponibilidade do diagnóstico radiológico de NC por TC sem contraste e/ou US renal na admissão, avaliação metabólica obtida a partir de pelo menos uma amostra de urina de 24 horas ou amostra isolada de urina (em casos de controle esfincteriano inadequado, em crianças pequenas), bem como dados bioquímicos e hormonais séricos. Foram coletados parâmetros demográficos e antropométricos, além de dados referentes à avaliação clínica inicial, com foco nas manifestações apresentadas na primeira consulta médica, como dor abdominal ou lombar, presença concomitante de NL, histórico de infecções urinárias de repetição (ITU) ou de procedimentos urológicos prévios, manifestações extrarrenais (surdez, fraqueza muscular, dificuldade de ganho pôndero-estatural), distúrbios eletrolíticos e comorbidades (hipertensão arterial e diabetes mellitus).

O protocolo padronizado de investigação consistiu na dosagem, em amostras de urina de 24 horas, de analitos como cálcio, sódio, potássio, citrato, oxalato, fósforo, ácido úrico, magnésio e cistina. Os critérios diagnósticos para distúrbios metabólicos urinários em adultos foram definidos conforme descrito em outras publicações^12^. Em crianças que coletaram amostras isoladas de urina, as alterações metabólicas foram diagnosticadas utilizando os valores de referência descritos por Hoppe e Higueras^13^. As medições do pH urinário em amostras isoladas de urina coletadas pela manhã, após jejum de 12 horas, realizadas simultaneamente com a dosagem do pH sanguíneo venoso e do bicarbonato plasmático, foram consideradas para o diagnóstico de ATRd. Na ausência de acidose espontânea, os pacientes foram submetidos a um teste de sobrecarga com cloreto de amônio (NH_4_Cl) para detectar formas incompletas de ATRd, conforme descrito anteriormente^4^. Outros parâmetros séricos determinados incluíram ureia, creatinina, potássio, magnésio, ácido úrico, cálcio, fósforo, hormônio da paratireoide (PTH), 25-hidroxivitamina D [25(OH)D] e 1,25-di-hidroxivitamina D [1,25(OH)_2_D]. A creatinina foi determinada por espectrometria de massa com diluição isotópica (IDMS), e a taxa de filtração glomerular estimada (TFGe) foi calculada utilizando a fórmula de *Schwartz* atualizada para crianças e a equação CKD-EPI (2021) para adultos. Os testes genéticos, quando disponíveis, consistiram principalmente em painéis gênicos ou no sequenciamento completo do exoma (WES). A interpretação da patogenicidade das variantes foi baseada nos critérios do ACMG (*American College of Medical Genetics and Genomics*)^14^, sendo então avaliada a correspondência com o fenótipo clínico.

### Análise Estatística

As distribuições das variáveis foram avaliadas pelo teste de *Shapiro–Wilk*. Os dados com distribuição normal foram expressos como média ± desvio padrão (DP), enquanto os dados com distribuição não normal foram expressos como mediana [intervalo interquartil]. As diferenças entre os grupos foram avaliadas utilizando o teste t de *Student* ou o teste de *Mann–Whitney*, de acordo com a distribuição da variável. As variáveis categóricas, apresentadas como n (%), foram comparadas por meio do teste do qui-quadrado. As análises foram realizadas utilizando o software JAMOVI, e valores de p < 0,05 foram considerados estatisticamente significativos.

## Resultados

Foram incluídos noventa e seis (96) pacientes, 66 adultos e 30 crianças, com diagnóstico radiológico de NC na admissão. A maioria dos pacientes adultos, 60 de 66 (91%), teve o diagnóstico de NC baseado em US, confirmada por TC, e 6 pacientes tiveram diagnóstico apenas por US. Entre as crianças, o diagnóstico radiológico baseou-se em US confirmada por TC em 14 de 30 casos (47%), em US isolada em 14 casos e exclusivamente em TC em 2 casos. As características clínicas e laboratoriais estão descritas na Tabela 1. Nos adultos, observou-se uma proporção significativamente maior de mulheres, maior associação com NL e menor mediana de TFGe em comparação com as crianças. O total de adultos com DRC a partir do estágio 2 superou significativamente o número de casos pediátricos. Diabetes e hipertensão arterial não foram observadas no grupo pediátrico. Embora aparentemente mais longo nos adultos, o tempo mediano até o diagnóstico não apresentou diferença estatisticamente significativa entre os grupos.

**Tabela 1:** Características clínicas e laboratoriais de todos os pacientes com nefrocalcinose radiológica na admissÃo

	< 18 anos (n = 30)	≥ 18 anos (n = 66)	p
Idade aos primeiros sinais/sintomas, anos	4,5 [0–8,5]	27,0 [18–35]	
Sexo feminino, n (%)	15 (50,0%)	49 (74,2%)	**0,02**
Atraso no diagnóstico, anos	4,5 [1,0–9,8]	8,0 [2,0–15,0]	0,14
Nefrolitíase associada	13 (43,3%)	48 (72,7%)	**0,01**
Procedimentos urológicos	8 (26,7%)	30 (45,5%)	0,08
Diabetes mellitus	0	4 (6%)	N/A
Hipertensão arterial	0	11 (17%)	N/A
Hematúria	11/23 (47,8%)	17/49 (34,7%)	0,29
Leucocitúria	9/23 (39,1%)	25/48 (52,1%)	0,34
TFGe (mL/min/1,73 m^2^)	106 [91–123]	85 [59–113]	**0,01**
DRC estágio 1	23 (77,0%)	32 (48,5%)	**0,01**
DRC estágios 2–5	7 (23,0%)	34 (51,5%)	**0,05**

Abreviaturas – TFGe, taxa de filtração glomerular estimada; DRC, doença renal crônica; N/A, Não aplicável.

Notas – Mediana [Intervalo interquartílico – IIQ] [25%–75%]; número de casos/número total de casos.

A Figura 1 mostra as principais manifestações observadas nos grupos pediátrico e adulto no momento do diagnóstico radiológico de NC. A porcentagem de casos assintomáticos não foi estatisticamente significativa entre crianças e adultos. No entanto, déficit de crescimento, perda auditiva neurossensorial e distúrbios eletrolíticos foram significativamente mais frequentes em crianças, enquanto a dor lombar ocorreu com maior frequência em adultos.

**Figura 1:** Principais características clínicas e laboratoriais de pacientes ao diagnóstico radiológico de NC.

### Etiologia da Nc Baseada no Fenótipo Inicial

A Tabela 2 apresenta as hipóteses diagnósticas de acordo com os dados clínicos, laboratoriais e de imagem renal obtidos durante a identificação da NC. Os casos foram classificados em três grupos: não sugestivos de origem genética, com suspeita de origem monogênica e de causa indefinida, quando não foi identificado nenhum fenótipo sugestivo.

**Tabela 2:** Etiologia da nefrocalcinose baseada no fenótipo inicial

	< 18 anos (n = 30)	≥ 18 anos (n = 66)	p
Fenótipo não sugestivo de origem genética (n = 25)			
Número de casos	**1/30 (3,3%)**	**24/66 (36,4%)**	**< 0,001**
HPTP	0	13 (54,0%)	
Induzido por drogas^#^	0	5 (21,0%)	
REM	1 (3,3%)	2 (8,3%)	
Hipoparatireoidismo pós-cirúrgico	0	2 (8,3%)	
ATRd completa + Síndrome de Sjogren	0	2 (8,3%)	
Fenótipo sugestivo de origem monogenética (n = 67)			
Número de casos	**29/30 (96,7%)**	**38/66 (57,6%)**	**< 0,001**
ATRd completa	10 (34,5%)	10 (26,2%)	
ATRd incompleta	3 10,4%)	5 (13,0%)	
HHRHc	3 (10,4%)	1 (2,6%)	
ADH	2 (6,8%)	0	
FHHNC	2 (6,8%)	0	
Doença de *Dent*	4 (13,8%)	0	
*Bartter*	0	3 (8,0%)	
*Lowe*	0	1 (2,6%)	
HP1	1 (3,5%)	1 (2,6%)	
Hipercalciúria isolada	4 (13,8%)	11 (29,0%)	
Hipocitratúria isolada	0	6 (16,0%)	
Causas indefinidas			
	**0**	**4/66 (6,0%)**	**N/A**

Abreviaturas – ATRd: Acidose tubular renal distal; FHHNC: Hipomagnesemia familiar com hipercalciúria e nefrocalcinose; HHRHc: Raquitismo hipofosfatêmico hereditário com hipercalciúria (carreador); ADH: Hipocalcemia autossômica dominante; REM: Rim esponjoso medular; HPTP: Hiperparatireoidismo primário; HP1: Hiperoxalúria primária tipo 1; N/A: Não aplicável.

Notas – ^#^Drogas incluindo furosemida, esteroides anabolizantes e corticosteroides.

Com base nos dados apresentados na Tabela 2, apenas uma criança apresentou um fenótipo não sugestivo de origem monogênica de NC, em comparação com 36,4% entre os adultos. Ainda assim, a proporção de adultos que exibiram um fenótipo sugestivo de doença monogênica foi considerável (57,6%).

A ATRd, incluindo as formas completa e incompleta, foi a causa predominante de NC em ambos os grupos. O HPTP foi o segundo diagnóstico mais prevalente entre os adultos, não sendo evidenciado em crianças, assim como a NC induzida por medicamentos. O rim esponjoso medular (REM) foi raramente diagnosticado, tanto em crianças quanto em adultos. Outras tubulopatias hereditárias raras foram identificadas predominantemente em crianças, embora algumas tenham sido diagnosticadas apenas mais tarde na vida adulta. A hiperoxalúria primária tipo 1 (HP1), uma doença metabólica genética grave, foi detectada em um caso de cada grupo, ambos os pacientes com DRC estágio 5. A hipercalciúria ou hipocitratúria isoladas foram mais frequentes em adultos. Em quatro adultos (6,0%), a causa da NC permaneceu indefinida.

### Testes Genéticos

Os resultados dos testes genéticos não estavam disponíveis em todos os casos, tendo sido realizados em 29 pacientes —16 crianças e 13 adultos — cujos achados estão apresentados na Tabela 3. O diagnóstico genético, baseado na suspeita de origem monogênica da NC, foi identificado em 55,2% dos casos pediátricos (16/29) versus 30,9% (13/42) dos adultos (p = 0,05). Na coorte total, a análise genética resultou em diagnóstico bem-sucedido em 22 dos 29 testes, alcançando uma taxa diagnóstica de 76,0%, confirmando o diagnóstico fenotípico em 65,5% (19/29) dos casos e modificando-o em 10,4% (3/29). No grupo pediátrico, o diagnóstico clínico foi confirmado em 68,7% (11/16) e modificado em 12,5% (2/16), resultando em uma taxa de sucesso de 81,2% (13/16). Entre os adultos, o teste confirmou a hipótese clínica em 61,5% (8/13) e a modificou em 7,7% (1/13), alcançando uma taxa de sucesso de 69,2% (9/13).

**Tabela 3:** Resultados dos testes genéticos

Pt.	Sexo (M/F) Idade (anos)	Suspeita clínica	Gene Zigosidade	Nucleotídeo; aminoácido	Classificação (ACMG)	Diagnóstico confirmado	Mudança do diagnóstico	Diagnóstico Genético final	Achados adicionais (gene estado/nucleotídeo proteína/classificação)
Coorte Pediátrica (n = 16)									
P1	M 7	ATRd	*SLC4A1* Het	c.2661_2680del; p.Ala888Leufs*2	Patogênica	X	----	ATRd	N/A
P2	M 10	ATRd	*SLC4A1* Het	c.2661_2680del; p.Ala888Leufs*2	Patogênica	X	----	ATRd	N/A
P3	F 14	ATRdi	*SLC4A1* Het	c.1825G>A;p.Gly609Arg	Patogênica	X	----	ATRdi	N/A
P4	M 5	ATRdi	Sem gene relacionado à NC	N/A	N/A	----	----	Ainda não definido	*GRHPR*/Het c.149C>T:p.Ala50Val/ VUS
P5	M 1	HHRH	*SLC34A3* Het	c.1217G>T;p.Gly406Val	Patogênica	X	----	HHRH (carreador)	N/A
P6	F 2	HHRH	*SLC34A3* Het	c.232G>A;p.Gly78Arg	VUS^#^	X	----	HHRH (carreador)	N/A
P7	F 7	HHRH	*SLC34A3* Het	c.1217G>T;p.Gly406Val	Patogênica	X	----	HHRH (carreador)	N/A
P8	M 4	*Dent*	*CLCN5* Hem ligado ao X	c.2151C>T; p.Arg718Thr	Patogênica	X	----	Doença de *Dent* tipo 1	N/A
P9	M 7	*Dent*	*OCRL* Hem ligado ao X	c.217_218delTT; p.Leu73Aspfs*2	Patogênica	X	----	Doença de *Dent* tipo 2	N/A
P10	F 4	*Dent*	Sem gene relacionado à NC	N/A	N/A	----	----	Ainda não definido	*CUBN*/Het composto c.8599G>C;p.Val2867Leu/VUS c.9053A>C;p.Tyr3018Ser/Provavelmente patogênica
P11	F 13	*Dent*	*SLC34A1* Het	c.460_480dup; p.Ile154_Val160dup	Patogênica	----	X	IIH tipo 2	N/A
P12	F 11	HI	*SLC34A1* Het	c.644G>A; p.Arg215Gln	Patogênica	----	X	IIH tipo 2	*SLC34A3*/Het c.1765G>A; p.Glu589Lys/Provavelmente benigna
P13	M 5	HP	*AGXT* Hom	exon 7 del	Patogênica	X	----	HP1	N/A
P14	M 0.8	FHHNC	*CLDN16* Hom	c.325_c918*?; E2_E5del	Patogênica	X	----	FHHNC	N/A
P15	F 6	ADH	*CASR* Het	c.2833T>C; p.Phe821Leu	VUS^#^	X	----	ADH	N/A
P16	F 17	HI	Sem gene relacionado à NC	N/A	N/A	----	----	Ainda não definido	*COL4A3/*Het c.4510T>C:p.Phe1504Le/ VUS
Coorte Adulta (n = 13)									
A1	F 18	ATRd	*SLC4A1* Het	c.2716G>T;p.Glu906*	Patogênica	X	----	ATRd	N/A
A2	F 23	ATRd	*SLC4A1* Het	c.2381A>G;p.Tyr794Cys	VUS^#^	X	----	ATRd	*SLC3A1*/Het c.1047C>A; p.Asp349Glu/VUS
A3	M 23	ATRd	*ATP6V1B1* Het	c.1181G>A;p.Arg394GIn	VUS^#^	X	----	ATRd	N/A
A4	M 31	ATRd	*ATP6V1B1* Het	c.1181G>A;p.Arg394GIn	VUS^#^	X	----	ATRd	N/A
A5	M 64	ATRd	*ATP6V1B1* Het	c.1181G>A;p.Arg394GIn	VUS^#^	X	----	ATRd	N/A
A6	M 27	ATRdi	*OXGR1* Het	c.566C>T;p.Ser189Leu	Provavelmente benigna (*novel*)	----	----	Ainda não definido	N/A
A7	M 41	HHRH	*SLC34A3* Het	c.232G>A;p.Gly78Arg	VUS	X	----	HHRH (carreador)	N/A
A8	F 21	HI	*SLC34A3* Het	c.413C>T / p.Ser138Phe	VUS	----	x	HHRH (carreador)	*GRHPR*/Het c296G>A; p.Arg99Gln / VUS
A9	M 37	HP	*AGXT* Het composto	c.508G>A;p.Gly170Arg - c.283G>A;p.Glu95Lys	Patogênica/ Provavelmente patogênica	X	----	HP1	N/A
A10	F 42	SB	*KCNJ1* Het	c.657C>A;p.Ser219Arg	Provavelmente patogênica	----	----	SB tipo 2 (carreador)	N/A
A11	F 35	HI	Negativo	N/A	N/A	N/A	N/A	Ainda não definido	N/A
A12	F 27	Sem doença relacionada à NC	Negativo	N/A	N/A	N/A	N/A	Ainda não definido	N/A
A13	F 29	Sem doença relacionada à NC	Negativo	N/A	N/A	N/A	N/A	Ainda não definido	N/A

Abreviaturas – Het: heterozigoto; hem: hemizigoto; hom: homozigoto; ATRd: Acidose tubular renal distal; ATRdi: Acidose tubular renal distal incompleta; FHHNC: Hipomagnesemia familiar com hipercalciúria e nefrocalcinose; HHRH: Raquitismo hipofosfatêmico hereditário com hipercalciúria; ADH: Hipocalcemia autossômica dominante; HI: Hipercalciúria idiopática; IHH tipo 2: Hipercalcemia idiopática infantil tipo 2; SB: Síndrome de *Bartter*; HP1: Hiperoxalúria primária tipo 1; VUS: variante de significado incerto; N/A: Não aplicável.

Notas – ^#^variante genética considerada relacionada à doença.

O diagnóstico foi revisado em dois casos pediátricos: uma menina de 13 anos, inicialmente com suspeita de doença de *Dent* (caso P11), e uma menina de 11 anos, com suspeita de hipercalciúria idiopática (caso P12). Ambas foram posteriormente diagnosticadas com hipercalcemia infantil idiopática tipo 2 (IIH tipo 2) por meio de teste genético. Além desses casos, o diagnóstico também foi revisado em uma paciente adulta, inicialmente diagnosticada com hipercalciúria idiopática (caso A8). O teste genético demonstrou uma variante de significado incerto (VUS) no gene *SLC34A3*, que explicava o fenótipo clínico, sendo, portanto, considerada uma variante relacionada à doença. De forma semelhante, nos casos P6 e A7, a variante no gene *SLC34A3*, classificada como VUS segundo os critérios da ACMG, também foi posteriormente considerada relacionada à doença. Em 24,1% (7/29) dos pacientes, o teste genético não contribuiu para a elucidação diagnóstica: quatro (4) adultos — incluindo dois pacientes (casos A12 e A13) com hipótese diagnóstica inicial indefinida; um caso de hipercalciúria idiopática (A11) sem variantes detectadas; e um caso de ATRd incompleta (A6), com identificação de uma variante provavelmente benigna no gene *OXGR1* — e três (3) pacientes pediátricos — incluindo um caso de suspeita de ATRd incompleta (P4), com identificação de uma variante heterozigótica de significado incerto em *GRHPR*; um caso de suspeita de doença de *Dent*, com variantes bialélicas em *CUBN*, que poderiam justificar a proteinúria, mas não a hipercalciúria; e um paciente com hipercalciúria idiopática, portador de uma variante patogênica heterozigótica em *COL4A3*.

### Diagnóstico Final

As distribuições individual e mediana de idade no momento do diagnóstico de NC, de acordo com as etiologias, estão apresentadas na Figura 2.

**Figura 2:** Etiologia da NC (ordem decrescente de frequência) de acordo com as idades individuais (o) do diagnóstico radiológico. Linhas horizontais (valores medianos).

Abreviaturas – Acidose Tubular Renal distal (ATRd); Hipercalciúria Idiopática (HCa); Hipocitratúria (HCit); Raquitismo Hipofosfatêmico Hereditário com Hipercalciúria carreador (HHRHc); Hipocalemia Autossômica Dominante (ADH); Hipomagnesemia Familiar com Hipercalciúria e Nefrocalcinose (FHHNC); Hipercalcemia Infantil Idiopática tipo 2 (IIH tipo 2); Rim Esponjoso Medular (REM); Hiperparatireoidismo Primário (HPTP); Hiperoxalúria Primária tipo 1 (PH1); Hipoparatireoidismo Pós-Cirúrgico (HPT); Não Determinada (ND).

## Discussão

As manifestações clínicas da NC podem ser heterogêneas, variando desde casos assintomáticos até alterações renais ou sistêmicas, cujas características dependem da etiologia subjacente e do momento do diagnóstico. De modo geral, as doenças renais monogênicas subjacentes são mais comuns na NC do que na NL^7,10,15^. Até onde sabemos, não há outras séries que descrevam as etiologias da NC em todas as faixas etárias no Brasil. Além disso, o país é subrepresentado em bancos de dados genéticos globais relacionados a diversas doenças^16^. Dessa forma, nosso objetivo foi identificar retrospectivamente as diferentes etiologias da NC e comparar os perfis clínicos e bioquímicos de pacientes adultos e pediátricos no momento do diagnóstico radiológico. Observou-se uma diferença significativa nas etiologias entre os dois grupos, exceto para a ATRd, que foi a principal causa em ambos. O HPTP foi evidenciado apenas em adultos, enquanto as doenças genéticas ocorreram predominantemente em crianças, grupo no qual os testes genéticos foram realizados com maior frequência.

Na presente série, os adultos apresentaram valores mais baixos de TFGe no momento do diagnóstico em comparação às crianças, possivelmente devido ao diagnóstico tardio da NC ou à maior prevalência de procedimentos urológicos prévios, embora nenhuma dessas diferenças tenha sido estatisticamente significante. As comorbidades e a associação com NL em adultos (72,7%), observadas também em outros estudos^17,18^, podem igualmente ter contribuído para a redução da função renal. Importante destacar que o déficit de crescimento e a surdez foram achados frequentes em crianças, provavelmente refletindo a maior prevalência de tubulopatias hereditárias nesse grupo19–21.

### Acidose Tubular Renal Distal (ATRd)

De acordo com os dados clínicos e laboratoriais, tanto as formas completas quanto as incompletas de ATRd foram as causas mais comuns de NC nesta coorte, identificadas em 43,3% das crianças e 22,7% dos adultos. Nossos resultados estão em concordância com um estudo anterior que identificou a ATRd como a causa mais frequente de NC em uma grande coorte de 375 pacientes^1^, sem distinção de idade. Na população pediátrica, as taxas de ATRd em casos de NC descritas na literatura variam de 5,6% a 34%^17,19,22^. Em adultos, um estudo antigo de Brenner et al.^23^, baseado em radiografias abdominais, identificou NC em 56% dos casos de ATRd. Porém, ainda são escassos os dados que especificam as taxas de ATRd como etiologia da NC em adultos^24^. Os achados do presente estudo reforçam a relevância de nossa ampla coorte de adultos com NC, evidenciando a ATRd como causa significativa.

Em relação ao espectro molecular, a ATRd foi o principal diagnóstico genético em nossa coorte (8/22; 36,4%), abrangendo todos os genes canônicos associados (*SLC4A1*, *ATP6V0A4* e *ATP6V1B1*). Frequências semelhantes de ATRd foram relatadas em séries envolvendo adultos^25^ e crianças^26^, enquanto taxas significativamente menores foram descritas por outros autores^10,21,27,28^. Como vários desses estudos foram realizados em centros terciários de referência, diferenças populacionais e nas etapas de investigação pré-laboratorial podem ter contribuído para a variação nas taxas diagnósticas e nas distribuições etiológicas observadas.

No presente estudo, apesar da forte concordância entre fenótipo e genótipo, reconhecemos que o teste genético deve ser recomendado a todos os pacientes com suspeita clínica de ATRd primária, a fim de aprimorar o prognóstico e o manejo, incluindo a avaliação de manifestações extrarrenais e o rastreamento familiar, considerando que a herança pode ser autossômica recessiva ou dominante^24,29^. Por exemplo, o paciente A4 (caso-índice; Tabela 3) apresentava uma variante heterozigótica no gene *ATP6V1B1* (*c.1181G>A; p.Arg394Gln*), associada a uma forma muito grave de NC e ATRd. A análise de segregação identificou a mesma variante em seu irmão (A3) e em seu pai (A5), ambos com fenótipos semelhantes, enquanto os testes genéticos foram negativos em sua mãe e em outro irmão, que não apresentavam manifestações clínicas ou NC. Entretanto, defeitos de acidificação renal e NL em portadores heterozigotos já foram previamente descritos na subunidade B1 desse gene^30,31,32^. Assim, embora essa variante tenha sido inicialmente classificada como VUS segundo os critérios da ACMG, a correlação com o fenótipo sugere tratar-se de uma variante relacionada à doença.

### Hiperparatireoidismo Primário (HPTP) e outras Etiologias Específicas do Adulto

O HPTP surgiu como a segunda etiologia mais comum entre os adultos (54%), em concordância com relatos anteriores^33,34^. Em contraste, não foram identificados casos no grupo pediátrico, o que ressalta as diferenças relacionadas à idade na etiologia da NC. O REM, terceira causa mais comum mencionada por Shavit et al.^1^, foi menos frequente em nossa coorte. Essa discrepância pode refletir limitações diagnósticas, uma vez que a confirmação por urografia excretora ou tomografia computadorizada com contraste não foi realizada de forma sistemática.

### Variantes Genéticas em *SLC34A1* e *SLC34A3*

Outro achado genético relevante desta coorte brasileira foi a alta taxa de variantes heterozigóticas relacionadas à doença nos genes *SLC34A1 e SLC34A3* (31,8%), que codificam, respectivamente, os cotransportadores sódio-fosfato NaPi-2a e NaPi- 2c. Diversos outros autores relataram variantes nesses genes em 10%^25^, 11,1%^10^, 23,9%^28^, 35,3%^21^ e 60%^5^ dos casos, em associação com hipercalciúria e NC. Identificamos variantes heterozigóticas no gene *SLC34A3* (Tabela 3) em quatro pacientes (P5, P6, P7 e A7), reforçando sua potencial contribuição para os fenótipos de NC/NL, mesmo quando na forma de VUS. A variante *SLC34A3* c.413C>T; p.Ser138Phe, identificada no caso A8 e previamente descrita em casos de raquitismo hipofosfatêmico hereditário com hipercalciúria^20,35,36^, demonstrou redução na atividade do cotransportador de fosfato dependente de sódio, sugerindo seu papel patogênico. De forma semelhante, as variantes *SLC34A3* c.232G>A; p.Gly78Arg, classificadas como VUS nos casos P6 e A7, foram consideradas relacionadas à doença com base na correlação fenotípica, conforme descrito anteriormente^37^. Em relação à modificação do diagnóstico final, a detecção molecular de variantes no gene *SLC34A1* nos pacientes P11 e P12 (inicialmente suspeitos de doença de *Dent* e hipercalciúria idiopática, respectivamente) alterou a conduta clínica, uma vez que aproximadamente 50% dos pacientes respondem à terapia com suplementação de fosfato, o uso de tiazídicos é contraindicado e o prognóstico renal difere quanto à progressão para DRC^37^.

### Outras Etiologias e o Papel dos Testes Genéticos

Entre outras causas de NC, HP1 e hipoparatireoidismo foram identificados em ambos os grupos. A identificação desses diagnósticos reforça a necessidade de uma investigação rápida e minuciosa, uma vez que algumas dessas condições podem levar à deterioração acelerada da função renal. A HP1 é um exemplo de doença genética grave que, se não tratada, resulta em desfechos renais desfavoráveis^6,38^, especialmente considerando que atualmente há um tratamento específico disponível, baseado em RNA de interferência^39^.

Em resumo, poucas séries publicadas avaliaram especificamente a NC e suas etiologias. Além disso, a maioria das coortes internacionais geralmente não apresenta dados clínicos, laboratoriais e genéticos da NC no momento do rastreamento^5,10,21,25,26,27,28,40^, analisando-a em conjunto com a NL, o que dificulta comparações com o presente estudo. Considerando especificamente os casos de NC isolada ou associada à NL, a taxa de testes genéticos positivos observada neste estudo foi de 76,0%, em comparação com 33,3% a 84,0% nas séries publicadas. Entre as crianças, a presença de doença monogênica foi de 81,2%, valor semelhante aos 84% relatados por Huang et al.^26^, embora superior aos 40% descritos por Braun et al.^27^ e Gefen et al.^5^. Entre os adultos, a taxa de doença monogênica associada à NC foi de 69,2%, em comparação com os 83,3% descritos em uma amostra menor e mais recente, composta por 12 pacientes adultos^25^.

No entanto, comparações entre diferentes coortes estão sujeitas a vieses decorrentes de diferenças nos padrões de encaminhamento, critérios de inclusão e metodologias analíticas (como o uso de painéis genéticos versus exoma completo, profundidade de sequenciamento, detecção de CNVs — variações no número de cópias, e atualizações nos critérios da ACMG), fatores que podem modificar tanto o rendimento diagnóstico quanto a aparente contribuição de genes específicos. A estrutura populacional também exerce influência: nossa coorte altamente miscigenada, composta por indivíduos de origens europeia, africana e indígena, altera as frequências alélicas e revela variantes pouco catalogadas, o que dificulta a interpretação dos achados^11^. Portanto, análises informadas pela população, a elaboração de mapas regionais de referência das etiologias de NC e o acesso equitativo a testes genéticos abrangentes são essenciais.

### Implicações Diagnósticas e Prognósticas

Em pacientes com forte suspeita de doença monogênica, especialmente quando familiares apresentam o mesmo fenótipo, quando a condição é potencialmente modificável e passível de tratamento específico, ou quando há manifestações extrarrenais que justifiquem uma investigação, o teste genético deve ser priorizado sempre que possível, pois pode estabelecer um diagnóstico definitivo e orientar o tratamento direcionado, como ilustrado anteriormente. Além disso, a análise molecular fornece subsídios importantes para o aconselhamento genético. No entanto, considerando que os testes genéticos ainda não estão amplamente disponíveis em muitos centros que atendem esses pacientes, este estudo sugere que a implementação de um protocolo racional de investigação clínica e laboratorial pode auxiliar na definição de indicações mais precisas para sua realização. Embora as doenças monogênicas tenham sido suspeitas em quase todas as crianças, a proporção de adultos com fenótipo sugestivo de doença monogênica ainda foi expressiva (57,6%), especialmente considerando o elevado número de pacientes com HPTP nesta coorte. Essa observação indica que o uso de testes genéticos em adultos deve ser mais frequente do que o observado atualmente.

### Limitações

As limitações do presente estudo incluem seu desenho retrospectivo e de centro único, a ausência de alguns valores laboratoriais nos prontuários médicos e a indisponibilidade de testes genéticos para todos os pacientes. Além disso, não foi aplicada correção para múltiplas comparações, em razão da heterogeneidade dos diagnósticos etiológicos em cada grupo analisado.

## Conclusões

Este estudo confirmou que a NC é frequentemente um sinal de doença renal ou sistêmica subjacente, não representando um diagnóstico etiológico em si, e está associada a alta morbidade, incluindo o risco de progressão para DRC. Os achados reforçam que a presença de NC fortemente sugere uma doença genética subjacente com envolvimento renal, especialmente em crianças, mas também evidenciam que muitos casos em adultos podem ter base monogênica, o que justifica a realização de testes genéticos também nesse grupo. Diante do acesso limitado aos testes genéticos em nossa população, provavelmente devido ao custo e à disponibilidade restrita no sistema público de saúde, uma avaliação clínica e laboratorial estruturada pode auxiliar na definição mais precisa das indicações de testagem genética em todas as faixas etárias.

## Disponibilidade de Dados

Todos os dados estarão disponíveis mediante solicitação.
